# Dermatologic manifestations of multisystem inflammatory syndrome in children during the COVID-19 pandemic^☆^

**DOI:** 10.1016/j.abd.2022.08.003

**Published:** 2022-11-16

**Authors:** Leyla Baykal Selçuk, Arzu Ferhatosmanoğlu, Zeynep Gökçe Gayretli Aydın, Deniz Aksu Arıca, Osman Yeşilbaş

**Affiliations:** a Karadeniz Technical University School of Medicine, Department of Dermatology and Venerology, Trabzon, Turkey; b Karadeniz Technical University, Faculty of Medicine, Division of Pediatric Infectious Diseases, Trabzon, Turkey; c Karadeniz Technical University, Faculty of Medicine, Division of Pediatric Intensive Care Unit, Trabzon, Turkey

**Keywords:** Child, COVID-19, Mucous membrane, SARS-CoV-2, Systemic inflammatory response syndrome

## Abstract

**Objectives:**

multi-system ınflammatory syndrome in children (MIS-C) is an immune-mediated process that develops after infections like SARS-CoV-2. The authors aimed to reveal the mucocutaneous findings of patients diagnosed with MIS-C at presentation and evaluate the frequency of these mucocutaneous findings and their possible relationship with the severity of the disease.

**Methods:**

A prospective study was conducted of 43 children admitted to a tertiary hospitals between January 2021 and January 2022 who met Centers for Disease Control and Prevention criteria for MIS-C.

**Results:**

43 children (25 [58.1%] male); median age, 7.5 years [range 0.5‒15 years]) met the criteria for MIS-C. The most common symptom was cutaneous rash 81.4%, followed by gastrointestinal symptoms 67.4%, oral mucosal changes 65.1%, and conjunctival hyperemia 58.1%. The most common mucosal finding was fissured lips at 27.9%, diffuse hyperemia of the oral mucosa at 18.6%, and strawberry tongue at 13.9%. Urticaria (48.8%) was the most common type of cutaneous rash in the present study’s patients. The most common rash initiation sites were the trunk (32.6%) and the palmoplantar region (20.9%). The presence or absence of mucocutaneous findings was not significantly associated with disease severity.

**Study limitations:**

The number of patients in the this study was small.

**Conclusions:**

The present study’s prospective analysis detected mucocutaneous symptoms in almost 9 out of 10 patients in children diagnosed with MIS-C. Due to the prospective character of the present research, the authors think that the characteristic features of cutaneous and mucosal lesions the authors obtained will contribute to the literature on the diagnosis and prognosis of MIS-C.

## Introduction

Coronavirus Disease-2019 (COVID-19) is a respiratory infection caused by the recently identified Severe Acute Respiratory Syndrome Coronavirus-2 (SARS-CoV-2). Multi-system Inflammatory Syndrome in Children (MIS-C) is an immune-mediated process that develops after infections like SARS-CoV-2; It presents with multi-organ involvement and elevation in inflammatory markers.^1^, ^2^, ^3^

There is some evidence that MIS-C is a postviral immune-mediated reaction. The occurrence of MIS-C 3‒5 weeks after the COVID-19 infection and the high percentage of IgG seropositivity support this situation.^1^, ^2^, ^3^, ^4^, ^5^ Most children do not display typical clinical manifestations of COVID-19 infection before developing MIS-C.

There is no specific, well-defined characteristic of the skin and mucosal lesions in literature due to the variability of MIS-C patterns. In this study, the authors aimed to reveal the mucocutaneous findings of patients diagnosed with MIS-C at the presentation and evaluate the frequency of these mucocutaneous findings and their possible relationship with the severity of the disease.

## Method

Forty-five patients who applied to the Faculty of Medicine, Farabi Hospital with MIS-C diagnosis between January 2021 and January 2022 were included in the present study. One patient was evaluated as having scarlet fever and one as Crimean-Congo Hemorrhagic Fever during follow-up, and therefore were excluded from the study.

According to the Centers for Disease Control and Prevention (CDC) diagnostic criteria,^6^ 43 patients with a definite diagnosis of MIS-C were included in this study. The MIS-C definition provided by the CDC was used in this study, which considers the following criteria: 1) An individual aged < 21 years presenting with fever (temperature 38.0 °C for 24 h, or report of subjective fever lasting 24 h); 2) Laboratory evidence of inflammation, including, but not limited to, one or more of the following: an elevated C-Reactive Protein (CPR), Erythrocyte Sedimentation Rate (ESR), fibrinogen, procalcitonin, D-dimer, ferritin, Lactic Acid Dehydrogenase (LDH), or Interleukin 6 (IL-6), elevated neutrophils, reduced lymphocytes, and low albumin; 3) Evidence of a clinically severe illness requiring hospitalization, with multisystem (two or more) organ involvement (cardiac, renal, respiratory, hematological, gastrointestinal, dermatological, or neurological); 4) A lack of an alternative plausible diagnoses, and 5) Positivity for current or recent SARS-CoV-2 infection by RT-PCR, serology, or antigen test, or exposure to a suspected or confirmed COVID-19 case within the 4-weeks prior to the onset of symptoms. The MIS-C definition provided by the CDC was summarized in the Online Supplementary Table S1. All patients underwent a complete dermatological physical examination at the presentation by the same dermatologist, and their rashes were followed up prospectively. The age, gender, contact or history of COVİD-19, and the severity of COVİD-19 in the family and the participants who gave consent to the study were recorded. Rash origin, involvement sites, and rash type (urticarial rash, maculopapular lesions, livedoid/necrotic lesions, pseudopernio, vesicular rash) were recorded.

Oral mucosal findings (strawberry tongue, fissured lip, cheilitis, Nagayama spots [erythematous macules], mucocutaneous ulceration, diffuse hyperemia of the oral mucosa, aphthous stomatitis) were recorded. Whether there is an erosion of the genital mucosa, history of conjunctival injection in the eyes, eyelid edema, nail symptoms, and hair findings were examined.

According to the CDC diagnostic criteria, MIS-C severity was divided into three groups mild, moderate, and severe, and the treatment protocols recommended in the guidelines were started.^7^ Disease severity classification is determined by the Vasoactive Inotropic Score (VIS), the degree of respiratory support, and evidence of organ injury.^8^ Mild cases have no vasoactive requirement, minimal respiratory support, and minimal signs of organ injury. In contrast, moderate cases have a VIS ≤ 10, significant supplemental oxygen requirement, and mild or isolated organ injury. Severe cases have a VIS > 10, non-invasive or invasive ventilatory support, and moderate or severe organ injury, including moderate to severe ventricular dysfunction.^9^ The blood value parameters of the patients at the time of diagnosis (White blood cell count, absolute lymphocyte count, d-dimer, procalcitonin, C-Reactive Protein [CRP], ferritin, pro-Brain Natriuretic Peptide [pro-BNP], troponin, sodium, albumin), the treatments they are receiving, the length of stay in the hospital and intensive care unit was recorded.

Ethics approval for this study was obtained from the local ethics committee.

### Statistical analysis

SPSS 23.0 statistical package program was used in the analysis of the data. Descriptive statistics of evaluation results; numbers and percentages are given for categorical variables, mean, standard deviation, minimum and maximum are given for interval variables. The conformity of the interval variables to the normal distribution was examined with the One-Sample Kolmogorov-Smirnov test. Comparisons of measurement data between independent groups; ANOVA and Student-*t*-Test were used when the normal distribution condition was met, and Kruskal-Wallis and Mann-Whitney *U* tests were used when they were not met. The Chi-Square test was used to analyze the differences between the ratios of categorical variables in independent groups. The statistical significance level was accepted as p < 0.05.

## Results

Of 43 patients diagnosed with MIS-C, 18 (41.9%) were female and 25 (58.1%) were male. The median age of the patients is 7.5 (0.5‒15) years old. The onset of MIS-C was observed at a median of 4 (1‒12) weeks after COVID-19 exposure. The sociodemographic and clinical characteristics of the patients are summarized in Table 1.

**Table 1:** Sociodemographic and clinical characteristics of the patients.

Age, years median (IQR)	7.5 (0.5‒15)
Gender, male (n %)	25 (58.1%)
Patients according to age group, n (%)	
<1 yr	1 (2.3%)
1‒4 yr	7 (16.3%)
5‒9 yr	20 (46.5%)
10‒14 yr	14 (32.6%)
15‒20 yr	1 (2.3%)
Underlying conditions	
Healthy	42 (97.7%)
Cystoperitoneal shunt	1 (2.3%)
Known COVİD-19 exposure, n (%)	35 (81.4%)
The time of COVİD-19 exposure before MIS-C onset, median weeks	4 (1‒12)
The severity of COVİD-19 infection	
Asymptomatic	32 (74.4%)
Mild	11 (25.6%)
The severity of COVİD-19 infection in the family	
Asymptomatic	14 (32.6%)
Mild	22 (51.2%)
Moderate	7 (16.3%)
SARS-CoV-2 testing with positive results	
Nasopharyngeal SARS-CoV-2 PCR	4 (9.3%)
Anti-SARS-CoV-2 immunoglobulin G	39 (90.7%)
Cutaneous symptoms, n (%)	35 (81.4%)
Gastrointestinal symptoms, n (%)	29 (67.4%)
Mucosal symptoms, n (%)	28 (65.1%)
Eye symptoms, n (%)	28 (65.1%)
Cardiovascular symptoms, n (%)	20 (46.5%)
Respiratory symptoms, n (%)	13 (30.2%)
Neurologic symptoms, n (%)	6 (14%)

The most common symptom was cutaneous rash 81.4%, followed by gastrointestinal symptoms 67.4%, oral mucosal changes 65.1%, conjunctival injection 58.1%, cardiovascular symptoms 46.5%, respiratory symptoms 30.2%, and neurologic symptoms 14%, respectively.

The polymerase chain reaction tests for SARS-CoV-2 were positive for 4 (9.3%) patients, and the results of SARS-CoV-2 immunoglobulin G tests were positive for 39 (90.7%) patients. 42 (97.7%) of the patients were completely healthy before infection, and 1 (2.3%) had a shunt.

**Figure 1:**
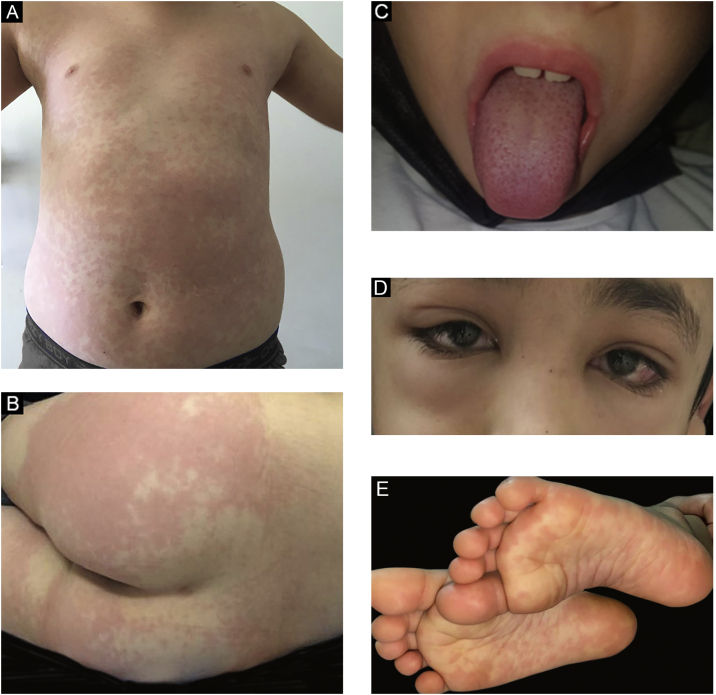
(a) Maculopapular rash on the trunk. (b) Erythematous urticarial plaque on the gluteal skin. (c) Strawberry tongue. (d) Periorbital edema and conjunctival injection. (e) Bilateral plantar erythematous macules.

In the present study, a median one day after the first symptom of MIS-C (fever, abdominal pain), the patients developed a mucocutaneous rash. There wasn’t any mucocutaneous involvement in 3 (6.9%) of the patients. Skin rash did not develop in 8 (18.6%) patients, and in 14 (32.6%) patients, the first site of the rash was the trunk. The mucocutaneous findings of the patients are summarized in Table 2. Some samples of clinical images are shown in Figs. 1a, 1b, 1c, 1d, and 1e. Maculopapular rash on the trunk is seen in Fig. 1a, erythematous urticarial plaque on the gluteal skin in Fig. 1b, strawberry tongue in Fig. 1c, periorbital edema and conjunctival injection in Fig. 1d, bilateral plantar erythematous macules in Fig. 1e, respectively.

**Table 2:** The mucocutaneous findings of the patients.

Mucocutaneous involvement, n (%)	40 (93%)
Cutaneous involvement, n (%)	35 (81.4%)
Cutaneous rash type, n (%)	
Urticarial lesions	21 (48.8%)
Maculopapular	11 (25.6%)
Livedoid rash	5 (11.6%)
Pseudopernio	1 (2.3%)
Cutaneous rash initiation site, n (%)	
Trunk	14 (32.6%)
Palmoplantar	9 (20.9%)
Lower extremity	6 (14%)
Genital	3 (7%)
Upper extremity	2 (4.7%)
Face	1 (2.3%)
Mucosal involvement, n (%)	28 (65.1%)
Fissured lips	12 (27.9%)
Cheilitis	10 (23.3%)
Diffuse hyperemia of the oral mucosa	8 (18.6%)
Strawberry tongue	6 (13.9%)
Herpes labialis	3 (6.9%)
Genital erosion	0
Eye involvement, n (%)	28 (65.1%)
Conjunctival injection	25 (58.1%)
Periorbital edema	4 (9.3%)
Symmetrical edema of the hands and feet	4 (9.3%)
Periungual desquamation, n (%)	2 (4.7%)

Laboratory findings, treatment throughout hospitalization, and outcomes of children with MIS-C are summarized in Table 3.

**Table 3:** Laboratory findings, treatment throughout hospitalization, and outcomes of children with MIS-C.

Laboratory results, median (IQR)	
WBC count (cells × 10^3^/μL)	9.33 (2.58‒28.14)
Absolute lymphocyte count (cells × 10^3^/μL)	0.85 (0.13‒3.84)
C-reactive protein (mg/L)	170 (11.5‒375)
D-dimer (mg/L)	3.41 (0.18‒36.48)
Albumin (g/L)	30.2 (19.5‒43.4)
Ferritin (μg/L)	327.2 (23.2‒11981)
Procalcitonin (μg/L)	3.88 (0.12‒185)
Pro BNP (ng/L)	1538 (9.62‒39880)
Troponin (ng/L)	9.39 (3‒800.6)
Sodyum (mEq/L)	133 (126‒143)
Treatment, n (%)	
Acetylsalicylic acid	39 (90.7%)
Intravenous immunoglobulin	33 (76.7%)
Systemic corticosteroids	35 (81.4%)
Plasmapheresis	1 (2.3%)
Clinical outcome, n (%)	
MIS-C severity, n (%)	
Mild	24 (55.8%)
Moderate	9 (20.9%)
Severe	10 (23.3%)
Hospitalization in the ICU, n (%)	19 (44.2%)
Days of hospitalization, median	7 (1‒53)
Days of ICU, median	3 (1‒7)
Oxygen	12 (27.9%)
Invasive mechanical ventilation	3 (6.9%)
ECMO	1 (2.3%)
Vasoactive drugs	20 (46.5%)
Death	2 (4.7%)

In the present study, mucocutaneous involvement was found in 66.7% of patients under five years of age, while mucocutaneous involvement was found in all patients over five years of age (p = 0.007). The mean total hospital stay under five years of age was 12.89 ± 15.431; the average length of stay in the ICU is 3.00 ± 1.414. The mean full length of hospital stay above five years of age was 7.47 ± 3,395; the average length in ICU was 3.38 ± 2.13. There was no statistically significant difference between patients younger than five years old and over five years old in total hospital stay (respectively p = 0.462, p = 0.133).

Comparisons and p values between mild and moderate/severe groups according to MIS-C severity are summarized in Table 4.

**Table 4:** Comparisons of the mucocutaneous lesions in mild and moderate/severe MIS-C groups.

	Mild MIS-C	Mod.-sev. MIS-C	p-value
> 5 years old, n (%)	20 (58.8%)	14 (41.2%)	0.477
Gender, male ‒ n (%)	12 (48%)	13 (52%)	0.366
Mucocutaneous involvement, n (%)	22 (55%)	18 (45%)	1.000
Cutaneous involvement, n (%)	19 (54.3%)	16 (45.7%)	1.000
Cutaneous rash type, yes ‒ n (%)			
Urticarial	10 (47.6%)	11 (52.4%)	0.453
Maculopapular	7 (63.6%)	4 (36.4%)	0.728
Livedoid rash	4 (80%)	1 (20%)	0.363
Pseudopernio	0	1 (100%)	0.442
Cutaneous rash initiation site, n (%)			
Trunk	11 (78.6%)	3 (21.4%)	a
Palmoplantar	4 (44.4%)	5 (55.6%)	a
Lower extremity	3 (50%)	3 (50%)	a
Genital	0	3 (100%)	a
Upper extremity	1 (50%)	1 (50%)	a
Face	0	1 (100%)	a
Mucosal involvement, n (%)	16 (57.1%)	12 (42.9%)	1.000
Mucosal rash type, n (%)			
Fissured lips	8 (66.7%)	4 (33.3%)	0.583
Cheilitis	4 (40%)	6 (60%)	0.295
Diffuse hyperemia of the oral mucosa	6 (75%)	2 (25%)	0.270
Strawberry tongue	3 (50%)	3 (50%)	1.000
Herpes labialis	0	3 (100%)	0.079
Eye involvement, n (%)	11 (45.8%)	13 (54.2%)	a
Conjunctival injection, n (%)	12 (48%)	13 (52%)	a
Symmetrical edema of hands and feet, n (%)	1 (25%)	3 (75%)	0.306
Periungual desquamation	0	2 (100%)	0.189
Hospitalization in the ICU, n (%)	4 (21.1%)	15 (78.9%)	**<0.001**

a Not calculated.

The most powerful aspect of the present study is that the authors followed the patients prospectively on a daily basis and thus, we were able to present a wide range of MISC-related mucocutaneous findings. In addition, all patients in the present study had the same ethnic origin, which made it a more homogeneous group in terms of genetic influence. However, some limitations should be noted. The authors couldn't take a skin biopsy from the cutaneous lesions. This was the most important limitation of the present study.

## Dıscussıon

In the present study, the most common symptom was cutaneous rash 81.4%, followed by gastrointestinal symptoms 67.4%, oral mucosal changes 65.1%, conjunctival injection 58.1%, cardiovascular symptoms 46.5%, respiratory symptoms 30.2%, and neurologic symptoms 14%. Pouletty et al.^9^ showed mucocutaneous involvement at 94%, and diffuse skin rash at 81%, and Toubiana et al.^10^ showed polymorphous skin rash in 76% in MIS-C. In the present study, the frequency of mucocutaneous involvement (93%) and cutaneous inflammation (81.4%) was observed at similar rates to the literature. The data obtained in the present study and other studies are summarized in Table 5.^2^, ^5^, ^7^, ^11^, ^12^, ^13^, ^14^ The first site of the rash was the trunk (32.6%), and the second most common was the palmoplantar region (20.9%). Urticaria (48.8%) was the most common type of cutaneous rash in the studied patients. In literature different from the authors’ findings, Yuksel et al. showed that maculopapular rash was the most common elementary lesion in 7 cases (41.2%).^13^

**Table 5:** Summary of data obtained in the present study and other studies.

Study/patient (n)	Our study (43)	Young TK, et al.^11^ (35)	Kaushik S, et al.^12^ (33)	Riollano-Cruz M, et al.^14^ (15)	Feldstein LR, et al. (186)^2^	Torres JP, et al.^5^ (27)	Jonat B, et al.^7^ (54)	Yuksel S, et al.^13^ (17)
Retro/prospective (R/P)	P	R	R	R	R + P	R + P	R + P	P
Age, years median	7.5 (0.5‒15)	2 (0.2‒17)	10 (6‒13)	12 (3‒20)	8.3 (3.3‒12.5)	6 (0‒14)	7 (0.8‒20)	8 (3.5‒13.5)
Gender, male, n (%)	25 (58.1)	19 (54)	20 (61)	11 (73)	115 (62)	14 (52)	25 (46)	7 (41.1)
Systemic symptoms								
Mucocutaneous symptoms, n (%)	40 (93)	29 (83)	7 (21)	NR	137 (74)	NR	NR	NS
Gastrointestinal symptoms, n (%)	29 (67.4)	31 (88.6)	NS	13 (87)	171 (92)	NS	45 (83)	13 (76.5)
Cardiovascular symptoms, n (%)	20 (46.5)	19 (54.3)	21 (63)	13 (87)	149 (80)	NR	NS	NR
Respiratory symptoms, n (%)	13 (30.2)	NS	NS	NR	131 (70)	NS	12 (22)	3 (17.6)
Neurologic symptoms, n (%)	6 (14)	NR	4 (12)	NR	NS	NR	22 (41)	3 (17.6)
Cutaneous rash, n (%)	35 (81.4)	28 (80)	14(42)	7 (47)	110 (59)	14(52)	41 (76)	NS
Urticarial lesions, n (%)	21 (48.8)	3 (9)	NR	NR	NR	NR	NR	NS
Maculopapular, n (%)	11 (25.6)	NR	NR	NR	NR	NR	NR	7(41.2)
Livedoid, n (%)	5 (11.6)	NR	NR	NR	NR	NR	NR	NR
Pseudopernio, n (%)	1 (2.3)	NR	NR	NR	NR	NR	NR	NR
Oral mucosal changes, n (%)	28 (65.1)	20 (57)	7 (21)	NR	78 (42)	11 (41)	NS	0
Fissured lips, n (%)	12 (27.9)	NR	NR	NR	NR	NR	NR	NR
Cheilitis, n (%)	10 (23.3)	NR	NR	NR	NR	NR	NR	NR
Diffuse hyperemia of the oral mucosa, n (%)	8 (18.6)	NR	NR	NR	NR	NR	NR	NR
Strawberry tongue, n (%)	6 (13.9)	8 (23)	NR	NR	NR	NR	NR	NR
Herpes labialis, n (%)	3 (6.9)	NR	NR	NR	NR	NR	NR	0
Genital erosion, n (%)	0	NR	NR	NR	NR	NR	NR	0
Eye involvement, n (%)	28 (65.1)	NS	NR	NR	NR	NR	NR	NR
Conjunctival injection, n (%)	25 (58.1)	21 (60)	12 (36)	4 (27)	103 (55)	13 (48)	31 (57)	9 (52.9)
Periorbital edema, n (%)	4 (9.3)	7 (20)	NR	NR	NR	NR	NR	NR
Symmetrical edema of the hands and feet, n (%)	4 (9.3)	14 (40)	NR	4 (27)	NS	NR	NR	NR
Periungual desquamation, n (%)	2 (4.7)	NR	NR	NR	NR	NR	NR	NR

NR, Non-Reported; NS, Non-Specified.

The authors observed oral mucosal involvement in 65.1% of the patients; the most common mucosal finding was fissured lips in 27.9%, diffuse hyperemia of the oral mucosa in 18.6% of the patients, and strawberry tongue in 13.9% of the patients. In Young et al.’s study, conjunctival injection (n = 21), palmoplantar erythema (n = 18), lip hyperemia (n = 17), chapped lips (n = 13), periorbital erythema and edema (n = 7), strawberry tongue (n = 8) and malar erythema (n = 6) were reported as the most common findings.^11^ In the present study, oral mucosal lesions were evaluated in detail in daily follow-ups compared to the literature; the frequency of oral involvement was higher.

The conjunctival injection was observed in 58.1% of patients, and periorbital edema was observed in 9.3%. Similar results were observed in the literature, with the rates of conjunctival injection at 57% and 55%.^2^, ^15^

Kawasaki disease is a vasculitis that affects small to medium vessels and usually affects infants and children under five. In more than 90% of cases, a diffuse maculopapular rash appears 3‒5 days after the onset of fever. An urticarial inflammation is rare. There may be erythema on the palms and soles, and periungual desquamation is usually observed 2 to 3 weeks after the onset of fever.^16^ However, unlike Kawasaki’s disease, MIS-C has been suggested to predominantly affect adolescents and children older than five years of age and be associated with more frequent cardiovascular involvement.^17^, ^18^, ^19^ Similar features were observed in the present study as well.

In the present study, 18 (41.9%) of 43 MIS-C patients were female, and 25 (58.1%) were male. The median age of the patients is 7.5 (0.5‒15). In the literature, Kaushik et al. showed that the median patient age was ten years; 61% of patients were male.^12^ Feldstein et al. reported a median age of 8.3 years; 62% were male.^2^ In contrast with the infantile age distribution of Kawasaki disease, MIS-C is predominantly a disease in older children and adolescents. Consistent with the literature in the present study, the male sex ratio was higher, and the median age was 7.5 years.

Although Black or Hispanic/Latin ethnicity was reported most frequently in most studies,^1^, ^12^ the present study revealed a more homogeneous group data as there was only one ethnicity (Turkish).

In the present study, systemic symptoms appeared a median of 4 (1‒12) weeks after exposure to COVID-19. A study by Belot et al.^20^ reported 4–5 weeks after the peak of COVID-19 cases.

COVID-19 in adults is typically more severe in patients with underlying conditions such as hypertension, diabetes mellitus, and other cardiovascular diseases, including cardiac and cerebrovascular disease.^21^^,^^22^ In contrast, more than half of MIS-C children seemed to have been previously healthy. In literature, comorbidity was not observed at 74%; in another study, 73% of the patients.^2^, ^5^ In the present study, 97.7% of patients were completely healthy and compatible with the literature.

In the present study, the median time to develop mucocutaneous complaints after the first symptom (fever, abdominal pain) was one day (range 1‒7 days). In the study of Torres et al., the median length of symptoms before admission was four days (range 2–9 days), and there was no data on the occurrence of mucocutaneous symptoms.^5^

The median hospitalization duration is 6.5 days in most studies.^1^ The median length of hospitalization was seven days among the patients who were discharged alive and five days among those who died.^2^ In the present study, the median length of hospital stay was seven days, and the median length of stay in the ICU was three days (1‒7).

## Conclusion

Dermatologic and mucocutaneous symptoms of MIS-C were commonly reported in the literature. However, studies evaluating skin and mucocutaneous findings in detail are limited in the literature. The prospective analysis detected mucocutaneous symptoms in almost 9 of 10 patients in children diagnosed with MIS-C. Due to the prospective course of the present research, the authors think that the characteristic features of the skin and mucosal lesions we obtained will contribute to the literature on the diagnosis and prognosis of MIS-C.

## Data availability statement

The data that support the findings of this study are available from the corresponding author.

## Financial support

None declared.

## Authors' contributions

Leyla Baykal Selcuk: Conception or design of the work; data collection; data analysis and interpretation; critical revision of the article; final approval of the version to be published.

Arzu Ferhatosmanoğlu: Conception or design of the work; data collection; data analysis and interpretation; critical revision of the article; final approval of the version to be published.

Zeynep Gökçe Gayretli Aydın: Data collection; critical revision of the article.

Deniz Aksu Arıca: Data collection; critical revision of the article.

Osman Yeşilbaş: Data collection; critical revision of the article.

## Conflicts of interest

None declared.

## References

[1] Guimarães D., Pissarra R., Reis-Melo A., Guimarães H. (2021). Multisystem inflammatory syndrome in children (MISC): a systematic review. Int J Clin Pract..

[2] Feldstein L.R., Rose E.B., Horwitz S.M., Collins J.P., Newhams M.M., Son M.B.F. (2020). Multisystem Inflammatory Syndrome in U.S. Children and Adolescents. N Engl J Med..

[3] Chiotos K., Bassiri H., Behrens E.M., Blatz A.M., Chang J., Diorio C. (2020). Multisystem inflammatory syndrome in children during the coronavirus 2019 pandemic: a case series. J Pediatric Infect Dis Society..

[4] Capone C.A., Subramony A., Sweberg T., Schneider J., Shah S., Rubin L. (2020). Characteristics, cardiac involvement, and outcomes of the multisystem inflammatory syndrome of childhood associated with severe acute respiratory syndrome coronavirus 2 infection. J Pediatrics..

[5] Torres J.P., Izquierdo G., Acuña M., Pavez D., Reyes F., Fritis A. (2020). Multisystem inflammatory syndrome in children (MIS-C): report of the clinical and epidemiological characteristics of cases in Santiago de Chile during the SARS-CoV-2 pandemic. Int J Infect Dis..

[6] emergency.cdc [Internet]. Centers for Disease Control and Prevention (CDC) Health advisory on multisystem inflammatory syndrome in children (MIS-C) associated with coronavirus disease 2019. Available from: https://emergency.cdc.gov/han/2020/han00432.asp. (Accessed on April 4, 2022).

[7] Jonat B., Gorelik M., Boneparth A., Geneslaw A.S., Zachariah P., Shah A. (2021). Multisystem Inflammatory syndrome in children associated with coronavirus disease 2019 in a children’s hospital in New York City: patient characteristics and an institutional protocol for evaluation, management, and follow-up. Pediatr Crit Care Med.

[8] Pouletty M., Borocco C., Ouldali N., Caseris M., Basmaci R., Lachaume N. (2020). Pediatric multisystem inflammatory syndrome temporally associated with SARS-CoV-2 mimicking Kawasaki disease (Kawa-COVID-19): a multicentre cohort. Ann Rheum Dis..

[9] McIntosh A.M., Tong S., Deakyne S.J., Davidson J.A., Scott H.F. (2017). Validation of the vasoactive-inotropic score in pediatric sepsis. Pediatr Crit Care Med..

[10] Toubiana J., Poirault C., Corsia A., Bajolle F., Fourgeaud J., Angoulvant F. (2020). Kawasaki-like multisystem inflammatory syndrome in children during the COVİD-19 pandemic in Paris, France: prospective observational study. BMJ..

[11] Young T.K., Shaw K.S., Shah J.K., Noor A., Alperin R.A., Ratner A.J. (2021). Mucocutaneous manifestations of multisystem inflammatory syndrome in children during the COVID-19 pandemic. JAMA Dermatol..

[12] Kaushik S., Aydin S.I., Derespina K.R., Bansal P.B., Kowalsky S., Trachtman R. (2020). Multisystem inflammatory syndrome in children associated with severe acute respiratory syndrome coronavirus 2 infection (MIS-C): a multi-institutional study from New York City. J Pediatrics..

[13] Yuksel S., Demirkan N.C., Comut E., Yilmaz M., Gurses D. (2022). Histopathological and clinical analysis of skin rashes in children with multisystem inflammatory syndrome associated with COVID-19. Am J Dermatopathol..

[14] Riollano-Cruz M., Akkoyun E., Briceno-Brito E., Kowalsky S. (2021). Multisystem inflammatory syndrome in children related to COVID-19: a New York City experience. J Med Virol..

[15] Lee P.Y., Day-Lewis M., Henderson L.A., Friedman K.G., Lo J., Roberts J.E. (2020). Distinct clinical and immunological features of SARS-CoV-2-induced multisystem inflammatory syndrome in children. J Clin Invest.

[16] Marchesi A., Jacobis I.T., Rigante D., Rimini A., Malorni W., Corsello G. (2018). Kawasaki disease: guidelines of the Italian Society of Pediatrics, part I ‒ definition, epidemiology, etiopathogenesis, clinical expression and management of the acute phase. Ital J Pediatr..

[17] Riphagen S., Gomez X., Gonzalez-Martinez C., Wilkinson N., Theocharis P. (2020). Hyperinflammatory shock in children during COVID-19 pandemic. Lancet.

[18] Belhadjer Z., Méot M., Bajolle F., Kraiche D., Legendre A., Abakka S. (2020). Acute heart failure in multisystem inflammatory syndrome in children (MIS-C) in the context of global SARS-CoV-2 pandemic. Circulation..

[19] Verdoni L., Mazza A., Gervasoni A., Martelli L., Ruggeri M., Ciuffreda M. (2020). An outbreak of severe Kawasaki-like disease at the Italian epicentre of the SARSCoV-2 epidemic: an observational cohort study. Lancet..

[20] Belot A., Antona D., Renolleau S., Javouhey E., Hentgen V., Angoulvant F. (2020). SARSCoV-2-related paediatric inflammatory multisystem syndrome, an epidemiological study, France, 1 March to 17 May 2020. Euro Surveill..

[21] Yonker L.M., Neilan A.M., Bartsch Y., Patel A.B., Regan J., Arya P. (2020). Pediatric severe acute respiratory syndrome coronavirus 2 (SARS-CoV-2): clinical presentation, infectivity, and immune responses. J Pediatrics..

[22] Kadkhoda K. (2020). COVID-19: an immunopathological view. mSphere..

