# Supplementary material for: Dermatologic manifestations of multisystem inflammatory syndrome in children during the COVID-19 pandemic
Source: An Bras Dermatol. 2022 Nov 16;98(2):168–75. doi: 10.1016/j.abd.2022.08.003 (PMC9666380; doi:10.1016/j.abd.2022.08.003)
Supplement: Supplementary file 1 [file mmc1.docx]

ABD-D-22-00291_Supplementary Material

**Supplementary Table 1** The MIS-C definition provided by the CDC.[6]

| 1) An individual aged < 21 years presenting with fever (temperature 38.0°C for 24h, or report of subjective fever lasting 24h) |
| --- |
| 2) Laboratory evidence of inflammation, including, but not limited to, one or more of the following: an elevated CRP, ESR, fibrinogen, procalcitonin, D-dimer, ferritin, LDH or IL-6, elevated neutrophils, reduced lymphocytes, and low albumin^a^ |
| 3) Evidence of a clinically severe illness requiring hospitalization, with multisystem (two or more) organ involvement (cardiac, renal, respiratory, hematological, gastrointestinal, dermatological, or neurological) |
| 4) A lack of alternative pausible diagnoses |
| 5) Positivity for current or recent SARS-CoV-2 infection by RT-PCR, serology, or antigen test, or exposure to a suspected or confirmed COVİD-19 case within the four weeks before the onset of symptoms |

^a^ C-Reactive Protein (CRP), Erythrocyte Sedimentation Rate (ESR), Lactic Acid Dehydrogenase (LDH), Interleukin-6 (IL-6).
